# Calcium-Based Nanoparticles Accelerate Skin Wound Healing

**DOI:** 10.1371/journal.pone.0027106

**Published:** 2011-11-02

**Authors:** Kenichiro Kawai, Barrett J. Larson, Hisako Ishise, Antoine Lyonel Carre, Soh Nishimoto, Michael Longaker, H. Peter Lorenz

**Affiliations:** 1 Hagey Laboratory for Pediatric Regenerative Medicine, Stanford University School of Medicine, Stanford, California, United States of America; 2 Department of Plastic and Reconstructive Surgery, Hyogo College of Medicine, Hyogo, Japan; University of California, Merced, United States of America

## Abstract

**Introduction:**

Nanoparticles (NPs) are small entities that consist of a hydroxyapatite core, which can bind ions, proteins, and other organic molecules from the surrounding environment. These small conglomerations can influence environmental calcium levels and have the potential to modulate calcium homeostasis *in vivo*. Nanoparticles have been associated with various calcium-mediated disease processes, such as atherosclerosis and kidney stone formation. We hypothesized that nanoparticles could have an effect on other calcium-regulated processes, such as wound healing. In the present study, we synthesized pH-sensitive calcium-based nanoparticles and investigated their ability to enhance cutaneous wound repair.

**Methods:**

Different populations of nanoparticles were synthesized on collagen-coated plates under various growth conditions. Bilateral dorsal cutaneous wounds were made on 8-week-old female Balb/c mice. Nanoparticles were then either administered intravenously or applied topically to the wound bed. The rate of wound closure was quantified. Intravenously injected nanoparticles were tracked using a FLAG detection system. The effect of nanoparticles on fibroblast contraction and proliferation was assessed.

**Results:**

A population of pH-sensitive calcium-based nanoparticles was identified. When intravenously administered, these nanoparticles acutely increased the rate of wound healing. Intravenously administered nanoparticles were localized to the wound site, as evidenced by FLAG staining. Nanoparticles increased fibroblast calcium uptake *in vitro* and caused contracture of a fibroblast populated collagen lattice in a dose-dependent manner. Nanoparticles also increased the rate of fibroblast proliferation.

**Conclusion:**

Intravenously administered, calcium-based nanoparticles can acutely decrease open wound size via contracture. We hypothesize that their contraction effect is mediated by the release of ionized calcium into the wound bed, which occurs when the pH-sensitive nanoparticles disintegrate in the acidic wound microenvironment. This is the first study to demonstrate that calcium-based nanoparticles can have a therapeutic benefit, which has important implications for the treatment of wounds.

## Introduction

Nanoparticles were first discovered almost two decades ago and were initially thought to be a type of “nanobacteria” [1], [2], [3], [4]. However, further research determined that nanoparticles were fundamentally different from typical living bacteria, and hence they were renamed, “nanoparticles” [5], [6], [7], [8], [9]. Studies ultimately revealed that nanoparticles were simply aberrant crystallizations of protein and minerals [6], [10], [11]. Recently, Young et al. proposed that nanoparticles can modulate environmental calcium levels and may play a role in calcium homeostasis [6], [7]. This ability to regulate calcium homeostasis is believed to have important physiologic and pathologic implications. Nanoparticles have been associated with many calcium-mediated disease processes, including cancer, kidney stone formation, and atherosclerosis [12], [13], [14], [15], [16], [17], [18].

Nanoparticles can be derived from many different source materials, but they are generally grown in the presence of serum and have a pleomorphic shape that ranges in size from 20–500 nm [3], [6]. The morphology, chemical composition, and physical properties of nanoparticles can be modulated by altering the protein and ionic composition of the culture medium [3], [4], [6], [10], [19]. Due to this intrinsic variability, it has been proposed that nanoparticles may represent a group comprising multiple distinct entities, each with unique characteristics [6]. However, one characteristic that is believed to be shared by nanoparticles is their ability to modulate calcium homeostasis [7].

Wound healing is a calcium-mediated process [20]. After cutaneous injury, a cascade of events occurs which ultimately results in tissue repair. Tissue repair is classically divided into an inflammatory phase, a granulation phase, and a scar-remodeling phase. A plethora of cells, enzymes, cytokines, hormones and ions are involved in this process. One of the most important ions involved in this process is calcium. Calcium is a key secondary messenger that is involved in several signaling cascades critical to wound healing [20]. Calcium influx into cells is known to regulate inflammatory cell infiltration, fibroblast proliferation and keratinocyte migration [21], [22]. Furthermore, calcium has an established role in the normal homeostasis of skin and is a modulator of keratinocyte proliferation and differentiation [20]. These features are highly relevant in the skin, which exists in a perpetual state of dynamic equilibrium with its environment.

Clinically, the direct topical application of calcium to chronic human wounds through calcium alginate dressings has been shown to be beneficial [23]. Multiple studies have shown that calcium alginate dressings increase the rate wound healing [24], [25]. The effect of calcium alginate dressings on cell proliferation is mediated by the release of ionized calcium into the wound bed. Increasing the ionized calcium concentration in the wound bed thus promotes improved healing [20], [26]. However, the greatest improvements occur in chronic wounds, such as venous ulcers [24]. This result suggests that topical calcium alginate is not an ideal calcium delivery system, as it does not effectively enhance acute wound healing.

We therefore wanted to address whether ionized calcium could be more effectively delivered to the acute wound bed. To do this, we engineered calcium-based nanoparticles (CNPs) that would readily disintegrate when exposed to an acidic pH. The pH of the acute wound microenvironment is known to be acidic, and ranges from a pH of 5–6 [27]. Since it is also known that calcium-phosphate minerals disintegrate in acidic conditions [28], we hypothesized that calcium-based nanoparticles could be designed to selectively disintegrate in the acidic wound microenvironment, and thereby cause a local release of ionized calcium which would enhance the repair process. To test this hypothesis, we designed *in vitro* and *in vivo* experiments to examine how our pH-sensitive calcium-based nanoparticles influence the repair process.

## Materials and Methods

### Ethics Statement

All animal procedures were conducted in accordance with NIH guidelines and with approval from Stanford University's Administrative Panel on Laboratory Animal Care (APLAC), protocol number 11048. APLAC guidelines for endpoint monitoring and humane termination were strictly followed. All animals used in this study were humanely euthanized with carbon dioxide prior to becoming moribund. Humane endpoints were used to reduce the duration and severity of pain and distress in experimental animals.

### Synthesis of nanoparticles

Nanoparticles were grown in 100 mm tissue culture dishes (#353803, BD Biosciences, Franklin Lakes, NJ) that were pre-coated with various different experimental coating solutions. The experimental plate-coating solutions were prepared by dissolving rat tail collagen (#11179179001, Roche, Indianapolis, IN) in sterile 0.2% acetic acid (pH 3.0) to obtain a final collagen concentration of 0.5 mg/ml. The ionic composition of the coating solution was then modified by systematically varying the concentration of CaCl_2_ and beta-glycerol-phosphate. In such a fashion, multiple different coating solutions were used for making different populations of nanoparticles. Then, 2.5 mls of coating solution was added to a 100 mm tissue culture dish. After a few moments, the coating solution was carefully decanted off, such that only a thin film of coating solution remained on the bottom of the dish. The dish was then dried and sterilized by leaving it uncovered in a fume hood under UV light for 12–16 hours. Each dish was washed once with PBS before growth media was added. The growth media consisted of alpha-MEM (#12571, GIBCO/Invitrogen, Carlsbad, California) supplemented with 20% FBS, penicillin/streptomycin (#15070-063, GIBCO/Invitrogen), and gentamicin (#15710-064, GIBCO/Invitrogen)]. Prior to culture, the growth media was oxidized by aeration and sterilized by passage through a 0.22 µm membrane five times. The nanoparticles were grown in an incubator at 37°C and 5% CO_2_. The media was changed every three days. Photos of nanoparticle cultures were taken daily with a digital camera (R8, RICHO, Tokyo, Japan) through a light microscope (CK40, Olympus, Tokyo, Japan).

### Determining the pH sensitivity of nanoparticles

Nanoparticles were synthesized in a 30 mm culture dish as described above and were considered ready for experimentation once the culture dish had reached near confluence. The culture dish was first washed with MilliQ water (Millipore, Billerica, MA) three times. Acidic solutions, ranging from pH 2.0 to pH 6.5, were prepared by adding HCl to MilliQ water. Then, the 30 mm culture dish containing the nanoparticles was fully submerged in a larger 150 mm culture dish that contained 200 ml of the appropriate acidic solution. Time lapse photographs of the nanoparticle cultures were taken using a digital camera (R8, RICHO, Tokyo, Japan) through a light microscope (CK40, Olympus, Tokyo, Japan). At each pH, the rate of nanoparticle disintegration was determined by measuring the time required for complete nanoparticle dissolution.

### Preparing nanoparticles for *in vivo* experiments

The nanoparticles were considered ready for *in vivo* experimentation once the culture dish had reached near confluence, which occurred approximately 2–4 weeks after their culture was initiated. The culture dish was first washed with PBS three times, 500 µl PBS was added, and the dish was scraped thoroughly. The nanoparticle solution was then centrifuged at 16,000 *g* for 30 min at 4°C and the resultant nanoparticle pellet was resuspended in 500 µl of normal saline (0.90% w/v of NaCl at 900 mOsm/L).

### Standardization of nanoparticle dose by electrical conductance

To determine the relative concentration of different nanoparticle preparations, an electrical conductance method was developed. A 100 µl aliquot of the CNP preparation was added to 1 ml of MilliQ water (pH 7.0) and the conductance was measured in µS/cm with a conductance meter (B-173, Horiba,Kyoto, Japan) at 25°C. The concentrations of different nanoparticle preparations were normalized based on the measured electrical conductance. The method was validated by measuring the electrical conductance of each nanoparticle preparation at different dilution factors to generate standard curves. The average nanoparticle preparation (resulting from one confluent 100 mm tissue culture dish) had an electrical conductance of approximately 13 µS/cm.

### Preparation of nanoparticles for Scanning Electron Microscopy (SEM) and Energy-Dispersive X-ray Spectroscopy (EDX)

The nanoparticles were prepared in 100 mm dish for SEM/EDX after 2–4 weeks of incubation. The culture dish was washed with PBS three times. Pure water (500 µl) was added and the dish was scraped thoroughly. An aliquot of the solution was deposited on a glass cover slide and dried in a fume hood overnight. The specimen was coated with gold. The nanoparticles were imaged with a scanning electron microscope (XL30 Sirion, FEI, Hillsboro, Oregon) and EDX was performed with the associated software (EDAX, Ametek, NJ).

### Open mouse wound model

8 week old female Balb/c mice were purchased from Charles Rivers Laboratories (Wilmington, MA). For cutaneous injuries, the mice were first anesthetized with isoflurane. The dorsum of each mouse was shaved and depilated. The skin was sterilized with betadine and 70% EtOH. All limbs were extended evenly so that the skin on the back became relaxed and symmetric. In order to make wound sizes as consistent as possible, the excision line was first traced with a 6 mm punch biopsy. The skin, including the panniculus carnosus, was carefully excised just above the myofascial layer with scissors. The wounds were washed with sterile saline and dressed with an anti-adhesive Telfa sheet (#7662, Covidien, Mansfield, MA) and Tegaderm (#1626, 3M, Maplewood, MN).

### Intravenous injection of nanoparticles

Immediately after the wounds were created, mice were injected via the tail vein with either 1) 100 µof the nanoparticle solution or 2) 100 µl of normal saline. Each experimental group consisted of 25 mice. Wound size was measured daily. Wound dressings were removed carefully with saline, so as not to change the wound size or shape. All limbs were extended evenly so that the skin on the back became relaxed and symmetric. A standard silicon ring was used as a reference frame for each photograph. Wound photographs were taken with a digital camera (D40, Nikon, Tokyo, Japan). The wound and reference ring areas were measured with Image J software (public software, NIH). The ratio of the wound area to the reference ring area was calculated, and the rate of wound closure was determined. Wounds were stained with picrosirius (#43665, Sigma-Aldrich, St. Louis, MO) red at day 7 and day 12 to assess the amount collagen deposition. Wounds were also stained using the von Kossa technique to identify calcium deposition.

### Intravenous injection of calcium chloride

Immediately after the wounds were created, mice were injected via the tail vein with either 1) 100 µl of calcium chloride (0.6 mg/mouse), 2) 100 µl of the nanoparticle solution, or 3) 100 µl of normal saline. Wound size was measured on days 0, 1, 2, 4, and 6. Wound closure rate was determined as described above for the nanoparticle injection experiments. Wound dressings were removed carefully with saline, so as not to change the wound size or shape. All limbs were extended evenly so that the skin on the back became relaxed and symmetric. A standard silicon ring was used as a reference frame for each photograph. Wound photographs were taken with a digital camera (D40, Nikon, Tokyo, Japan). The wound and reference ring areas were measured with Image J software. The ratio of the wound area to the reference ring area was calculated and the rate of wound closure was determined.

### Serum calcium level after intravenous injection of calcium-based nanoparticles and calcium chloride

Serum calcium levels were measured at 1 hr, 3 hr, 12 hr, 24 hr, 72 hr and 12 days after injection of either 1) 100 µl of the calcium-based nanoparticle solution, 2) 100 µl of calcium chloride (0.6 mg/mouse), or 3) 100 µl of normal saline. Mice were sacrificed and blood was collected from the aorta with a heparinized syringe (26 G needle). The blood was transferred to a 1.5 ml tube (on ice) and centrifuged at 2000 rpm for 10 min at 4°C. The upper serum layer was transferred to another tube (on ice). The serum calcium level was measured with a calcium assay kit (#Z5030014, BioChain, Hayward, CA), as per the manufacturer's instructions.

### Tracking intravenously injected calcium-based nanoparticles

In order to track CNPs after intravenous injection, FLAG (+) CNPs were synthesized. To synthesize FLAG (+) CNPs, FLAG peptide (F3290, Sigma-Aldrich, St. Louis, MO) was added to the nanoparticle growth medium (alpha-MEM, 20% FBS, Pen/Strep/Gent) to obtain a final FLAG peptide concentration of 100 mg/ml. The FLAG (+) CNPs were grown in 6 well plates that had been pre-treated with coating solution. A total of 2 ml of FLAG (+) medium was added to each well, and the medium was changed every three days. The FLAG (+) CNPs were considered ready for *in vivo* experimentation once the culture dish had reached near confluence, which occurred approximately 2 weeks after their culture was initiated. The culture dish was washed with PBS three times and scraped thoroughly. The FLAG (+) CNPs were centrifuged at 16,000 *g* for 30 min at 4°C, and then resuspended in normal saline. 100 µl of FLAG (+) CNPs were then injected via the tail vein into wounded 8 week old female Balb/c mice. Control mice were injected with either 100 µl of FLAG (-) CNPs or 100 µl of normal saline. Mice were sacrificed at 0 hours, 3 hours, 24 hours, 72 hours, 7 days, and 10 days. Wound tissue was harvested and soaked in 10% formaldehyde at 4°C overnight and embedded in paraffin. The tissue samples were cut to a thickness of 4 µm.

FLAG (+) CNPs were detected using a mouse monoclonal biotin-conjugated anti-FLAG antibody (F9291, Sigma-Aldrich, St. Louis, MO). After deparaffinization, the specimens were treated with blocking solution (PBS with 2% horse serum and 0.1% Tween 20) for one hour at room temperature. Antibody was diluted 1∶100 with the blocking solution, and specimens incubated with biotinylated antibody solution overnight at 4°C. Then, specimens were washed with the blocking buffer three times, and incubated with the ABC kit (#PK4000, Vector Laboratories, Burlingame, CA) for 30 min at room temperature. The specimens were washed again three times and incubated with DAB solution (#25985-50, Nacalai Tesque, Kyoto, Japan) for exactly 4 min. The specimens were then rinsed thoroughly with water and imaged.

### Topical application of calcium-based nanoparticles

Open mouse wounds were created on 8 week old female Balb/c mice, as described previously. The wounds were treated with a topical application of either 1) 100 µl of the calcium-based nanoparticle solution 2) 100 µl of calcium chloride (0.6 mg/mouse) or 3) 100 µl of normal saline. The calcium-based nanoparticles were applied directly to the wound bed. Calcium chloride and normal saline controls were applied in the same manner. Wound size was measured at 0, 1, 2, 4, 6 and 8 days. A standard silicon ring was used as a reference frame for each photograph. Wound photographs were taken with a digital camera (D40, Nikon, Tokyo, Japan). The wound and reference ring areas were measured with Image J software. The ratio of the wound area to the reference ring area was calculated.

### Assessing fibroblast proliferation after treatment with calcium-based nanoparticles

NIH3T3 cells were purchased from American Type Culture Collection (ATCC), Manassas, VA (CRL-1658). The cells were cultured in DMEM high glucose medium (#11995, GIBCO/Invitrogen, Carlsbad, CA) supplemented with 10% FBS and Penicillin/Streptomycin and grown at 37°C in 5% CO_2_. The cells were passaged with trypsin/EDTA (#25300, GIBCO/Invitrogen,) prior to confluence. The cells were seeded in 96 well plates (CLS3596, Costar/Sigma-Aldrich, St. Louis, MO) at concentrations of 5,000/well (50 µl/well). After incubation for 2 days, the cells were treated with CNPs. Calcium-based nanoparticles were prepared as described above and a pellet of CNPs from a 100 mm dish was suspended in 500 µl of medium (DMEM high glucose, 10% FBS, Pen/Step). CNPs were then suspended in medium at dilutions of 1∶1 to 1∶100. 50 µl of the CNP solution was added to each well. The treated and controlled wells were incubated for 24 hours. BrdU labeling was then performed to quantitate proliferation (#11647229001- BrdU (colorimetric) Cell Proliferation ELISA, Roche, Indianapolis, IN). After addition of BrdU, plates were incubated at 5% CO_2_ and 37°C for 2 hours. The 370 nm absorbance was measured as per the manufacturer's instructions.

### Treatment of Fibroblast Populated Collagen Lattice (FPCL) with calcium-based nanoparticles

NIH3T3 cells were cultured as described above. Trypsinized cells were mixed with 1 mg/ml of rat tail collagen dissolved in 3 mM HCl at a concentration of 50,000 cells/ml. 500 µl of the cell-collagen mixture was added to each well of a 24-well plate (#353847, BD, Franklin Lakes, NJ). The FPCL gel formed at 5% CO_2_ and 37°C for 2 hr. Then, 500 µl of the CNP solution (DMEM high glucose with 10% FBS and P/S at pH 7) was added to each well. The CNP-containing medium was replaced daily. The 24 well plate was scanned digitally (Scanjets 5370C and c7671b, Hewlett-Packard, Palo Alto, CA) and the size of the FPCL was measured daily and calculated with Image J software (NIH), normalizing to the day 0 value.

### Calcium uptake in fibroblasts after treatment with calcium-based nanoparticles

NIH3T3 cells were cultured as described above. The cells were seeded in poly-D-lysine coated 96 well plates (#354461, BD, Franklin Lakes, NJ) at a concentration of 5,000 cells/well (50 µl/well). After incubation for 2 days, the cells were treated with calcium-based nanoparticles from 1∶1 to 1∶100 dilution. The medium was replaced with 50 µl/well of medium (DMEM high glucose, 10% FBS, Pen/Step) and 50 µl of Fluo4 reagent (F10471, Fluo-4 Direct™ Calcium Assay Kit, Invitrogen, Carlsbad, CA). The Fluo-4 reagent was used according to the manufacturer's protocol. The cells were incubated at 5% CO_2_ and 37°C for 30 min and subsequently incubated at room temperature for 30 min in the dark. The level of fluorescence in each well was measured with a Cytofluor 2 plate-reader (PerSeptive Biosystems, Framingham, MA). The excitation and emission wavelengths were 492/20 and 528/30, respectively.

### Statistical Analysis

Data involving only two groups was analyzed with an independent sample two tailed *t-*test assuming unequal variances. When more than two experimental groups were compared, the data was analyzed using Dunnett's multicomparison test to compare data between individual experimental groups and the corresponding control. A p-value value of < 0.05 was considered to be statistically significant for all tests. All data presented graphically in figures is expressed as the mean ± SD.

## Results

### Nanoparticle formation

**Figure 1:**
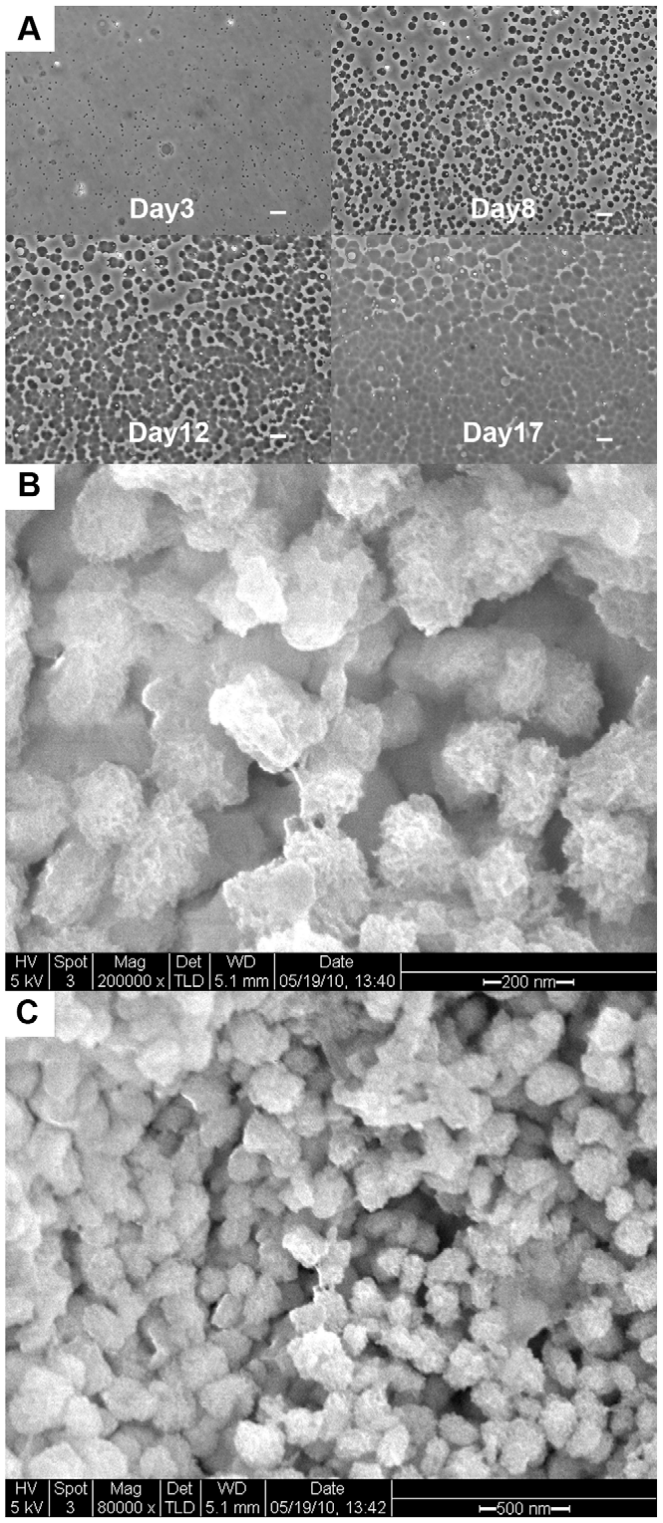
The growth and morphology of calcium-based nanoparticles in culture. (A) Small circular particles were visible on the culture plates starting at day 3. The particles grew in both size and number, and were pleomorphic. Over time, the particles began to coalesce, and by day 14 they were confluent. Scale bar  =  10 µm (B) SEM of calcium-based nanoparticles on day 14 at 200,000x. (C) SEM of calcium-based nanoparticles on day 14 at 80,000x

Nanoparticles formed on plate surfaces and were first visible as tiny circular-shaped particles at day 3 (Figure 1A). The particles grew in both size and number, and were pleomorphic. The particles were firmly attached to the collagenous coating solution on the dish surface. To confirm that the particles were not bacteria (or any type of living organism), the particles were cultured in 6% sodium hypochlorite overnight with UV radiation. The particles were stable in bleach and UV radiation (data not shown). Figure 1 (B and C) demonstrates that the size of the individual nanoparticles ranged between 50-200 nm *in vitro*. Over time, the individual small circular precipitates grew in size and became confluent on day 14.

### Disintegration of calcium-based nanoparticles occurs in a pH-dependent fashion

To assess the effect of environmental pH on the stability of calcium-based nanoparticles, near-confluent cultures of CNPs were treated with acid (HCl titrated to pH of 2.0–6.5). A relatively large volume of acid (200 ml) was used to treat the CNPs, in order to mitigate the pH buffering effect of the nanoparticles. Interestingly, the CNPs buffered the pH of the solution and maintained an equilibrium pH of 7.4 when 8 mL of acid solution was added to a near-confluent 100 mm dish of nanoparticles. However, the buffering capacity of CNPs was exceeded when 200 ml of acid was added. The rate of CNP disintegration was directly related to the pH of the environment. At a pH of 2.0, the CNPs completely disintegrated within 2 min. As the pH was increased, the rate of CNP disintegration decreased until a pH 5.5. No appreciable CNP disintegration occurred after 48 hours of incubation in pH 6.0 solution.

### Therapeutic effect of nanoparticles depends on culture conditions

**Figure 2:**
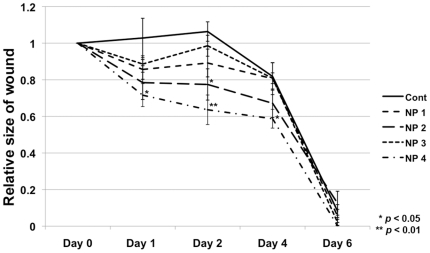
Therapeutic effect of nanoparticles depends on growth parameters. Four different populations of calcium-based nanoparticles (NPs) were synthesized (represented as NP 1 through NP 4). For each population, the concentration of calcium chloride and glycerol phosphate were varied in the coating material. NP 1: collagen (0.5 mg/ml). NP 2: collagen (0.5 mg/ml) + beta-glycerophosphate (5 mM). NP 3: collagen (0.5 mg/ml) + CaCl_2_ (2 mM). NP 4: collagen (0.5 mg/ml) + beta-glycerophosphate (5 mM) + CaCl_2._ These different nanoparticle populations were injected through the tail vein of wounded mice, and the rate of wound healing was assessed for each group. Each nanoparticle population had a different effect on wound healing. P-values were calculated using the Dunnett's test. The data is expressed as the mean ± SD.

The physical properties and therapeutic effect of nanoparticles were found to vary based on the ionic composition of the coating solution used. The size, shape, growth characteristics, and therapeutic effect of nanoparticles could be modulated by altering the concentrations of CaCl_2_ and beta-glycerol-phosphate in the coating solution. Figure 2 demonstrates how the rate of wound closure was affected by the ionic composition of the coating solution used to synthesize the nanoparticles. The nanoparticle population with the greatest effect on wound healing was synthesized using a coating solution that contained CaCl_2_ and beta-glycerol-phosphate at concentrations of 2 mM and 5 mM respectively. All subsequent experiments were performed using calcium-based nanoparticles that had been synthesized using the aforementioned coating solution.

### Ionic composition of calcium-based nanoparticles

**Figure 3:**
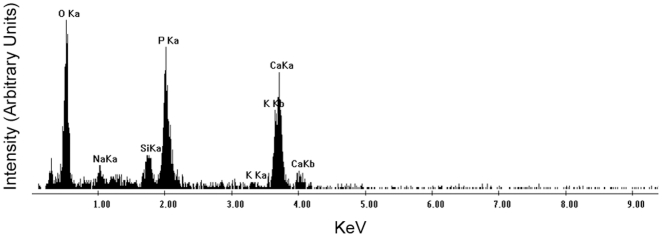
The ionic composition of calcium-based nanoparticles. SEM/EDX analysis demonstrates the ionic composition of our calcium-based nanoparticles. These nanoparticles represent a collection of charged ions including oxygen, sodium, silica, phosphorous, potassium, and calcium. The predominant ions are calcium, oxygen, and phosphate. Note: Ka represents the energy yield from the L → K electronic orbital transition and Kb represents the energy yield from the M → K electronic orbital transition.

Scanning electron microscopy (SEM) of the CNPs showed that their sized ranged from 50-200 nm. Energy dispersive x-ray (EDX) spectral analysis of these particles showed a high peak of calcium (Figure 3). The spectrum is also suggestive of the presence of phosphate, since there are high peaks of both phosphorous and oxygen. This data is consistent with the spectral analysis of other previously reported nanoparticles [6], [7].

### Intravenous injection of calcium-based nanoparticles accelerates wound healing and decreases scar formation in excisional mouse wounds

**Figure 4:**
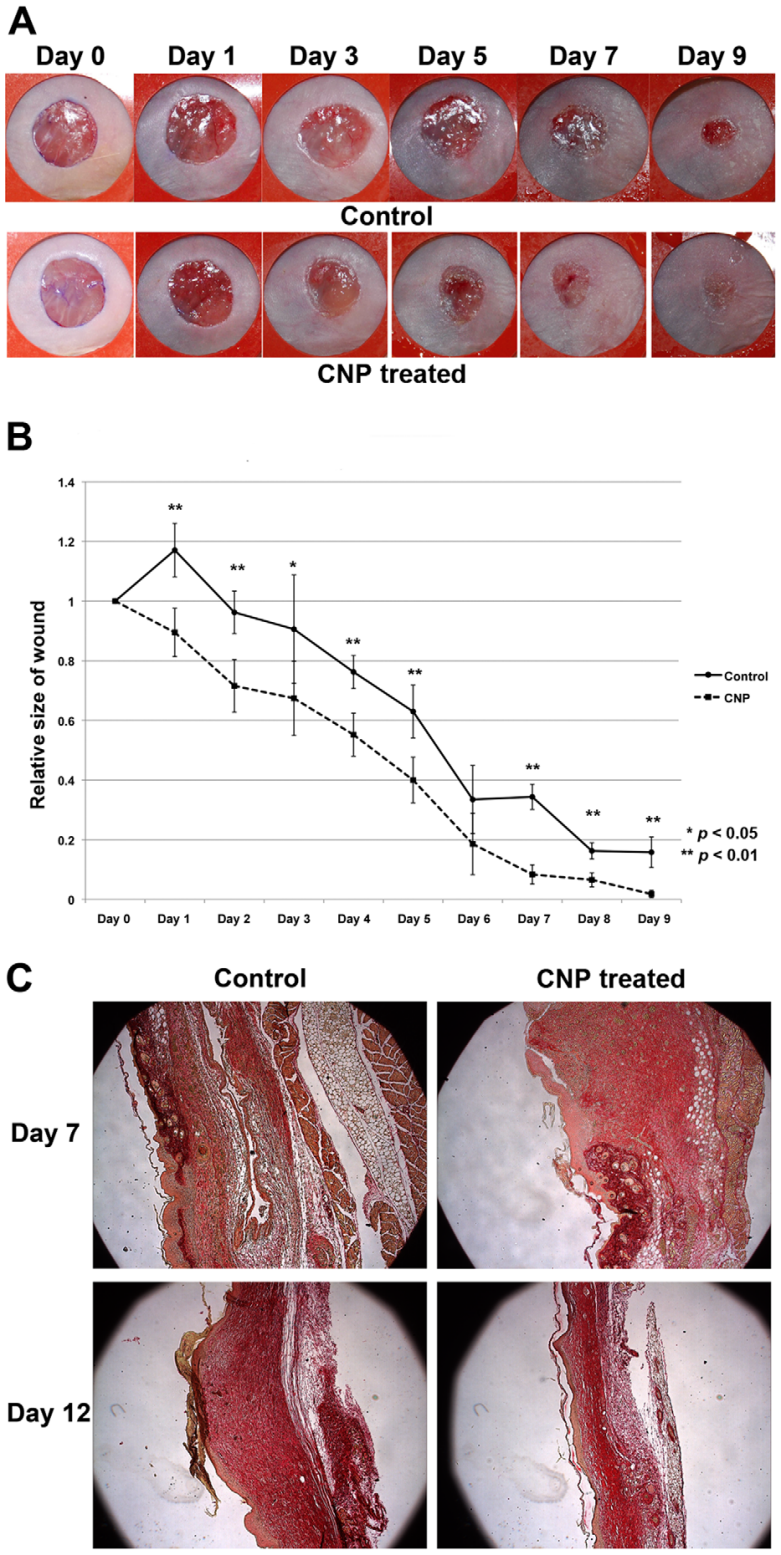
Intravenous injection of calcium-based nanoparticles accelerates healing of open mouse wounds. (A) Representative photographs of mouse cutaneous wounds injected with calcium-based nanoparticle (CNP) suspension, or saline control. Differential healing was observed at all five time points checked (N = 5 for each time point). (B) Quantification of the wound area in nanoparticle-and control-treated wounds from days 0-9. The data is expressed as the mean ± SD. P-values were calculated using the Student's t-test (two-tailed). (C) Histologic sections representing the degree of collagen deposition in nanoparticle-treated vs. control wounds. Sections were taken from the center of wounds and stained with picrosirius red at post-injury days 7 and 12. Collagen deposition was qualitatively more rapid in nanoparticle-treated wounds.

The effect of calcium-based nanoparticles on wound healing was investigated on open 8 mm full thickness excisional mouse skin wounds. Mice received a single injection of calcium-based nanoparticles at the time of injury. For the first 24 hours following injury, the rate of wound closure was found to be significantly faster in calcium-based nanoparticle treated mice. However, after 24 hours, the rate of wound closure was similar between CNP-treated and control mice (Figures 4A and 4B). Although the rate of wound closure approximated controls after 24 hours, the absolute wound size remained significantly smaller in CNP-treated mice at all time points. Wounds were stained with picrosirius red to assess the amount of collagen deposition. Sections were taken from the center of wounds. At day 7, CNP-treated mice had wounds with increased collagen deposition relative to controls. However, by day 12, CNP-treated mice had healed with less collagen deposition than controls (Figure 4C).

### Intravenous injection of calcium chloride accelerates wound healing while significantly increasing serum calcium levels

**Figure 5:**
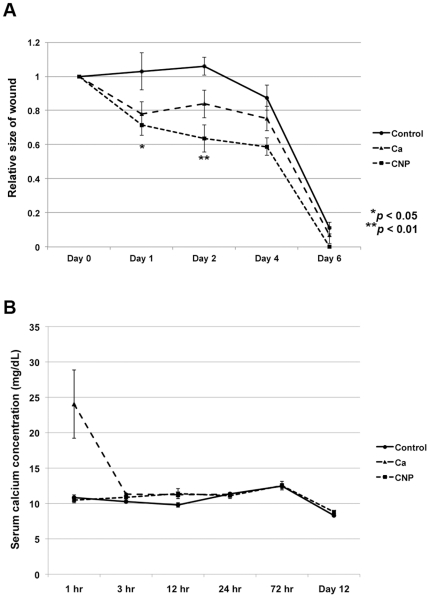
Intravenous injection of calcium chloride and calcium-based nanoparticles. (A) Wounded mice received an injection of either calcium chloride, calcium-based nanoparticles (CNPs) or saline via the tail vein. The rate of wound healing was assessed after treatment in each group. Both calcium chloride and CNP-treatment accelerated wound healing relative to controls. CNP-treatment significantly decreased wound area compared to calcium chloride treatment by day 2 (N = 4 per group). P-values were calculated using the Dunnett's test. The data is expressed as the mean ± SD. (B) Serum calcium levels of mice were measured after they received an injection of calcium chloride, calcium-based nanoparticles or saline. Serum calcium levels remained unchanged after CNP injection, but were significantly increased 1 hour after CaCl_2_ injection.

In order to determine if calcium alone would have the same wound-healing effect as calcium-based nanoparticles, mice received an intravenous injection of calcium chloride (0.6 mg/mouse) immediately following cutaneous injury. The rate of wound healing was found to be accelerated in mice receiving both calcium chloride and calcium-based nanoparticles (Figure 5A). However, by day 2 the rate of wound healing was found to be faster in mice receiving CNPs versus calcium chloride. Although the rate of wound healing was enhanced in both CNP-treated and calcium-treated mice, mice treated with intravenous calcium chloride experienced negative side effects due to hypercalcemia. All experimental mice treated with intravenous calcium chloride demonstrated significant muscle tetany that lasted for approximately 1 hour following treatment. Furthermore, approximately 20% of mice receiving intravenous calcium chloride died within minutes of treatment due to cardiac arrest. When the serum calcium levels were analyzed, it was found that mice receiving calcium chloride had total serum calcium levels that were approximately 2.5x greater than controls (Figure 5B). In CNP-treated mice, there was no evidence of negative side effects due to hypercalcemia. No experimental mice receiving CNPs displayed signs of muscle tetany, and no CNP-treated mice died following treatment. The serum calcium levels in CNP-treated mice were found to be within normal limits and approximated that of control mice (Figure 5B).

### Topical administration of calcium-based nanoparticles does not accelerate wound healing

**Figure 6:**
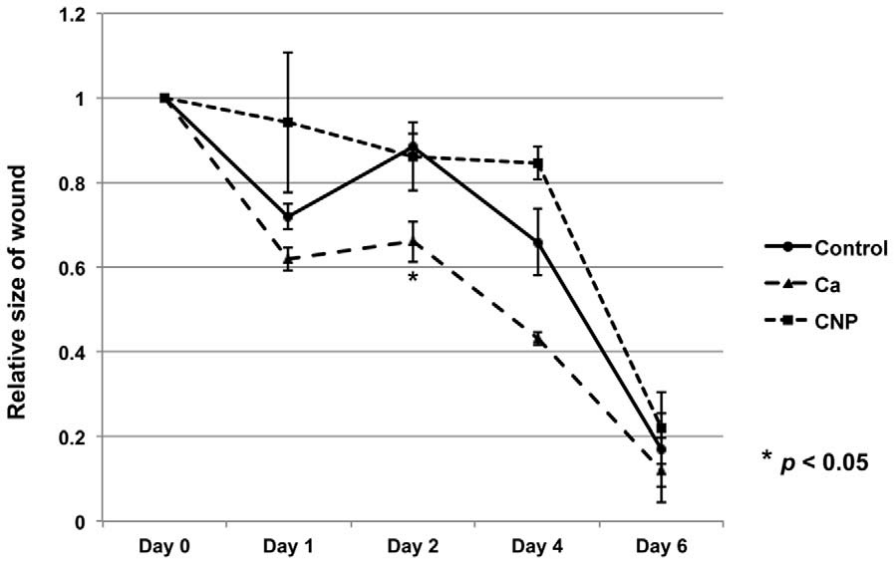
Topical administration of calcium-based nanoparticles does not accelerate wound healing. Wounded mice received a topical application of either calcium chloride, calcium-based nanoparticles (CNPs) or saline. Topically applied calcium-based nanoparticles did not enhance wound healing. However, topically applied calcium chloride significantly increased the rate of wound healing relative to controls. P-values were calculated using the Dunnett's test. The data is expressed as the mean ± SD.

In order to assess the effect of localized delivery of calcium-based nanoparticles, groups of wounded mice received a topical application of either CNPs, calcium chloride, or normal saline. Wounds were measured at 0, 1, 2, 4, and 6 days. Topical application of CNPs did not significantly change the wound healing rate (Figure 6). However, consistent with previous studies [20], [23], [24], [25], the direct topical application of calcium chloride accelerated the wound healing rate (Figure 6).

### Calcium-based nanoparticles increase fibroblast proliferation and contraction

**Figure 7:**
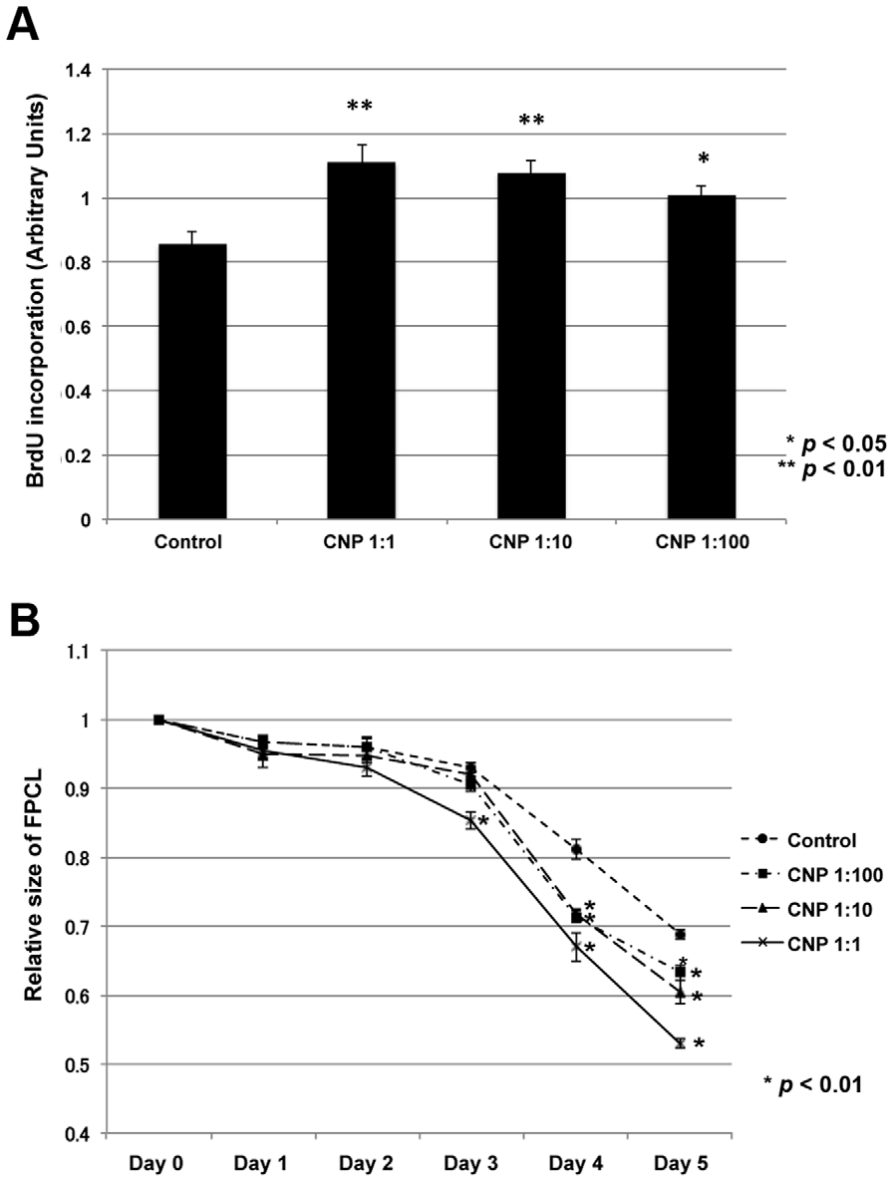
Calcium-based nanoparticles increase fibroblast proliferation and contraction. (A) BrdU incorporation after 12 hrs of growth with calcium-based nanoparticles (CNP) in NIH3T3 fibroblasts. CNP-treatment increased fibroblast proliferation rate, which was dependent on CNP concentration in a dose-dependent manner. (B) Fibroblasts were added to a collagen lattice to form a fibroblast populated lattice complex. The surface area of the FPCL reduced over time depending on CNP concentration. The amount of contractility is inversely proportional to FPCL size. CNP-treatment increased FPCL contraction. P-values were calculated using the Dunnett's test. The data is expressed as the mean ± SD.

In order to further investigate the mechanism by which nanoparticles accelerate wound repair, we investigated their effect on fibroblast cells (NIH3T3). Bromodeoxyuridine (BrdU) incorporation was performed to assess their effect on proliferation. Fibroblasts were incubated for 2 days, treated with nanoparticles from 1∶1 to 1∶100 dilution, and then incubated for 24 hours. Calcium-based nanoparticle treatment induced a ∼25% increase in BrdU incorporation at the highest CNP concentration (Figure 7A).

Fibroblast-populated collagen lattice (FPCL) was performed to determine the effects of CNP treatment on cellular contraction. NIH3T3 fibroblasts were cultured in a collagen lattice, and CNPs were added daily for 5 days. While the collagen lattice contracted with time, CNP treatment enhanced this contraction in a dose-dependent manner compared to controls (Figure 7B).

### Calcium-based nanoparticles are a vehicle for the delivery of calcium to the wound bed

**Figure 8:**
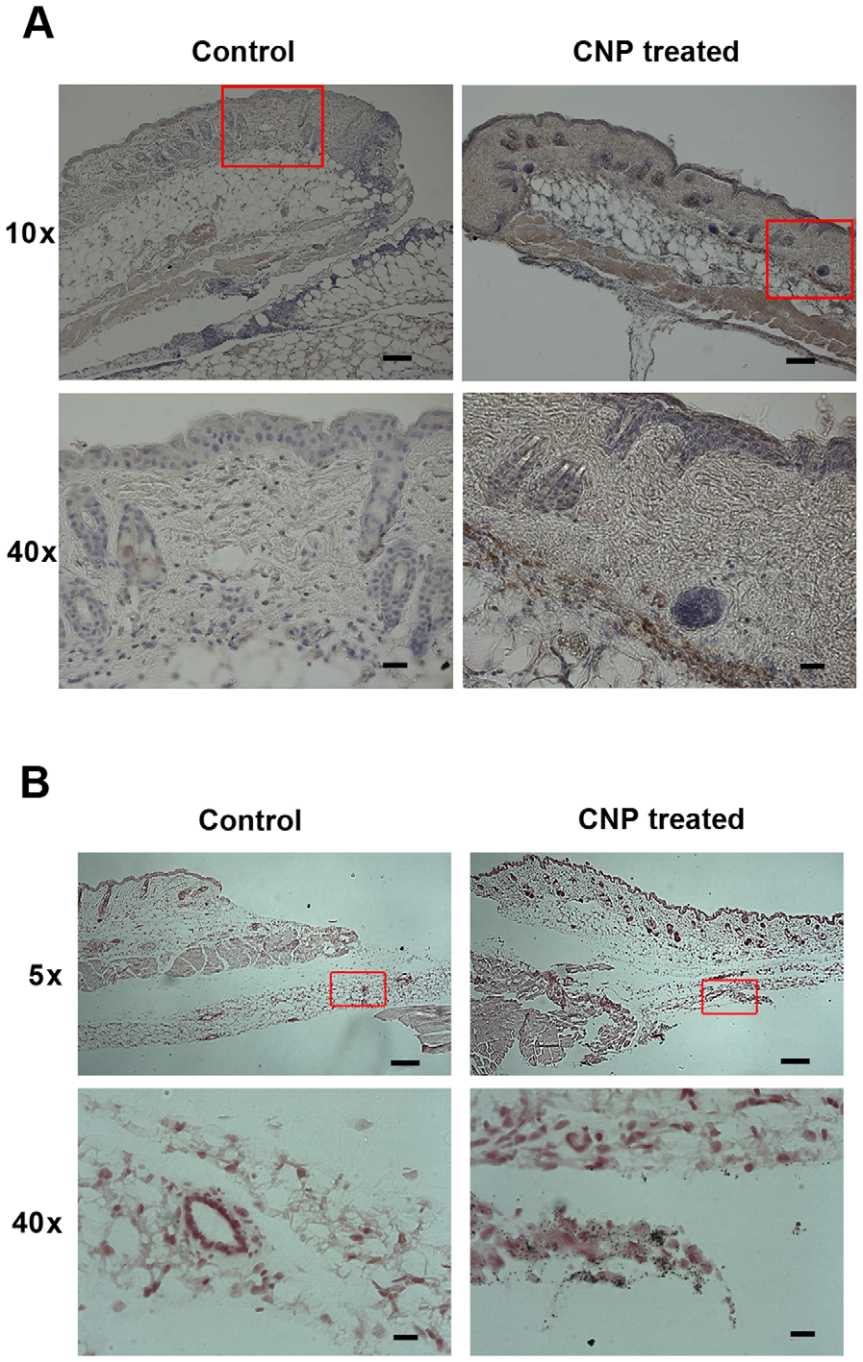
Calcium-based nanoparticles are a vehicle for the delivery of calcium to the wound bed. (A) Intravenously injected FLAG (+) calcium-based nanoparticles (CNPs) are detected using an anti-FLAG antibody on day 1. FLAG (+) cells are located in the epidermal, dermal, and panniculus carnosus muscle layers at the wound site. Scale bars for 10x images  =  100 µm, for 40x images  =  20 µm. (B) Von Kossa staining demonstrates calcium (black dots) in the deep wound bed of the CNP-treated mice. Scale bars for 5x images  =  200 µm; for 40x images  =  20 µm.

Due to the pH-sensitivity of our calcium-based nanoparticles, we hypothesized that CNPs accelerated wound healing by releasing calcium into the acidic wound microenvironment. This local release of calcium would then induce fibroblast proliferation and cause wound contracture, as we observed in our *in vitro* experiments. In order to test this hypothesis, nanoparticles were synthesized with an incorporated FLAG peptide. These FLAG (+) CNPs were then tracked using immunohistochemical assays. Immediately following cutaneous injury, mice received an intravenous injection of FLAG (+) CNPs. The wound tissue was harvested, and the FLAG peptide probed. Significantly more FLAG staining in the wound beds of mice treated with FLAG (+) CNPs, compared to mice treated with FLAG (-) CNPs, or normal saline occurred (Figure 8A). FLAG staining was most prominent in the deep muscle layer of the wound bed (Figure 8A). The FLAG stain was greatest at day 1, and then substantially decreased in intensity over subsequent days. The FLAG-positive CNPs appeared to localize specifically to sites of injury, as there was no evidence of FLAG stain in non-wounded skin tissue. Furthermore, increased calcium deposition in the deep wound bed of CNP-treated mice was demonstrated by von Kossa stain (Figure 8B).

**Figure 9:**
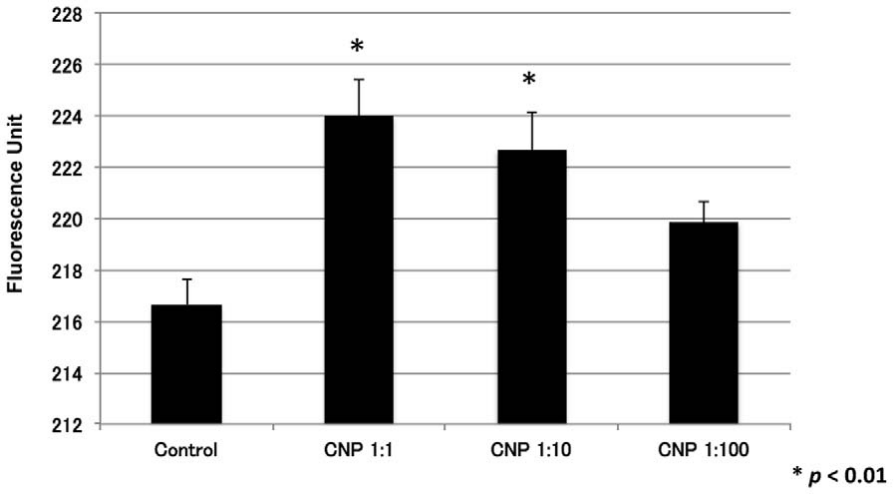
Calcium-based nanoparticle treatment increases fibroblast calcium uptake. Fibroblasts in culture showed increased calcium uptake in a dose dependent manner after calcium-based nanoparticle treatment. P-values were calculated using the Dunnett's test. The data is expressed as the mean ± SD.

In order to determine the ability of calcium-based nanoparticles to deliver calcium intracellularly, the fluorescence of Fluo4 AM was measured in NIH3T3 fibroblasts before and after treatment with nanoparticles. Fluo4 AM is a fluorescent calcium indicator and is used to measure Ca^2+^ levels inside living cells. Calcium-based nanoparticle treatment increased calcium influx in a dose dependent manner (Figure 9).

## Discussion

The results presented here, for the first time, indicate that nanoparticles may have a potential therapeutic benefit. Nanoparticles have been associated with various calcium-mediated disease processes, but there have been no reports suggesting that nanoparticles can potentially influence calcium homeostasis in a favorable manner. We reasoned that if nanoparticles can modulate environmental calcium levels as previously suggested [6], [7], then perhaps nanoparticles could be engineered to disintegrate and release calcium in an acidic environment. Nanoparticles could thereby selectively increase ionized calcium levels in acidic wound tissue. Our results demonstrate that calcium-based nanoparticles can be designed to release calcium as a function of pH, and when injected intravenously these calcium-based nanoparticles can accelerate acute cutaneous wound healing by causing wound contracture.

Nanoparticles were originally thought to be living organisms, but more recently they have been described as a group of abiotic entities with distinct chemical and physical properties [5]. In this study, the nanoparticle growth parameters were modified extensively in order to generate calcium-based nanoparticles that could effectively enhance wound healing. We found that the size, morphology, and growth characteristics of nanoparticles were highly dependent on the source materials and growth parameters used. We initially selected for nanoparticle populations that were microscopically similar to those previously described. Then, we selected for populations of nanoparticles that demonstrated pH sensitivity within the range of what is expected in the acute wound environment. Finally, we tested four different nanoparticle populations *in vivo*, and found that each had variable effects on wound healing (Figure 2). The nanoparticle population that was found to be most effective in wound healing was grown on a substrate that contained 2 mM CaCl_2_ and 5 mM beta-glycerol-phosphate. Our data demonstrates that the physiologic properties of nanoparticles can be altered by modulating growth parameters and culture conditions. Therefore, enhancement of the beneficial wound healing properties of calcium-based nanoparticles is likely possible with further modulation of growth parameters and culture conditions.

The acute wound microenvironment is known to be acidic and has a pH that ranges between 5-6 [27]. This temporary and localized acidosis is caused by an accumulation of lactic acid that results from cellular hypoxia at the wound margin combined with an increased metabolic oxygen demand during the healing process [27]. Since calcium-phosphate minerals dissolve in the presence of acidic conditions [28], we reasoned that nanoparticles could potentially function as a pH-based calcium delivery vehicle. We developed a population of calcium-based nanoparticles that readily disintegrated and released their calcium payloads when the environmental pH dropped below pH 5.5. By administering these pH-sensitive calcium-based nanoparticles intravenously, calcium could then be selectively deployed in acidic regions of the body, such as wound sites. This pH-based delivery system allowed for localized delivery of calcium, without any effect on total serum calcium levels.

Delivery of calcium to the wound bed enhances healing [20], [23], [24], [25]. An influx of calcium is an essential component of the repair process, and the addition of exogenous calcium can enhance wound healing. Calcium exerts its therapeutic effect on wound healing via multiple mechanisms and results in faster, more efficient repair. In the present study, we attempted to deliver ionized calcium to the wound bed using calcium-based nanoparticles. We administered these CNPs both topically and intravenously, and compared their effect on wound healing to that of calcium chloride. Consistent with previous studies [20], [23], [24], [25], we found that the topical application of calcium chloride increased the rate of wound healing. However, the topical application of CNPs did not accelerate wound healing. Presumably, the CNPs were not able to readily release their calcium payloads when applied topically. This suggests that the wound surface environment may not be conducive towards nanoparticle calcium-release. Another potential explanation for why topically applied nanoparticles did not accelerate wound healing relates to nanoparticle internalization into cellular compartments. Recently, internalization of nanoparticles into the cellular lysosomal compartment has been shown *in vitro* [29]. If internalization of nanoparticles into the cellular compartment is a mechanism responsible for nanoparticle-mediated effects, then perhaps nanoparticle internalization occurs more readily when they are delivered intravenously. That being said, the pharmacokinetics behind nanoparticle delivery deserves further investigation in order to better understand their mechanism of action.

Although intravenous calcium chloride treatment accelerated wound healing, significant calcium-related side-effects occurred. Intravenous calcium chloride caused dramatic elevations in the total serum calcium levels and this treatment was associated with significant morbidity and mortality. However, as opposed to treatment with intravenous calcium chloride, treatment with intravenous calcium-based nanoparticles rapidly accelerated wound healing but did not elevate serum calcium levels and did not result in any morbidity/mortality. These observations demonstrate the benefit of selectively delivering calcium to wound sites.

No significant difference in wound healing rates between intravenously administered calcium-based nanoparticles and topically applied calcium chloride were observed. Given the potential risks associated with intravenous administration of nanoparticles, it is not clear if treatment with calcium-based nanoparticles is superior to topically applied calcium chloride. However, we found that the physiologic effects of calcium-based nanoparticles can be dramatically changed by altering their growth parameters. By continuing to optimize their growth parameters and dosing schedule, we may be able to develop a nanoparticle-based calcium delivery system that outperforms topical calcium chloride and be therapeutic for acute wound healing. The present findings demonstrate that calcium-based nanoparticles can be used as a pH-based calcium delivery vehicle and can enhance wound healing, but further studies are needed to determine whether this system is superior to topical calcium chloride.

On a technical note, we developed a new method for determining the dose of nanoparticles given to our experimental animals. By measuring the electrical conductance of a nanoparticle preparation, the relative concentration could be determined. For our *in vivo* studies, each animal in the treatment group received 1 dose of nanoparticles. We defined a “dose” to be the amount of nanoparticles derived from one confluent 100 mm tissue culture dish. We found that the average dose was 100 μl of a nanoparticle solution that had an electrical conductivity of 13 µS/cm. Although there was some dish-to-dish variation in the concentration of nanoparticles, our final results were highly consistent across multiple different preparations of nanoparticles.

In future studies, we plan to vary the dose of calcium-based nanoparticles given in order to develop a dose-response curve. The rate and quality of wound repair may be further enhanced by administering larger or more frequent doses of CNPs. In the present study, we found that the rate of wound healing was only increased during the first 24 hours following treatment with CNPs, after which point the rate of wound healing paralleled that of controls. Experiments are planned to assess whether serial injections of CNPs can be used to maintain the increased rate of wound healing beyond 24 hours.

Although we observed no evidence of nanoparticle-related toxicity, this may change with higher or more frequent dosing. Previous animal studies have shown that intravenous injections of nanoparticles can induce an immune sensitizing response [30]. If immune sensitization were to occur after initial exposure to calcium-based nanoparticles, this may result in an adverse immune reaction upon subsequent exposure. Animals in our study received one dose of nanoparticles at the time of wounding, and were not subjected to a second dose in which a sensitization reaction could occur.

Furthermore, studies suggested that nanoparticles may lead to pathologic calcification, such as atherosclerosis [31]. However, this evidence has recently been called into question [32]. The use of γ-FBS has led to publications implicating nanoparticles in the pathogenesis of many diseases, and most of these studies were based on the underlying assumption that the gamma-irradiated serum used to culture nanoparticles was sterile, and thus any nanoparticle growth originated from the specific tissue sample under investigation. Martel and colleagues demonstrated that this underlying assumption is fundamentally flawed, and that non-irradiated serum produces nanoparticles that are equivalent to those produced using gamma-irradiated serum [32]. Therefore, the nanoparticles that presumably were derived from the tissue sample under investigation were actually derived from a combination of antigens present in the γ-FBS, in addition to antigens present in the experimental tissue sample. The use of γ-FBS to culture nanoparticles predisposes to introducing organic components from foreign species, which may confound experimental findings. In our study, no attempt was made to culture nanoparticles from experimental tissue samples, as this would potentially lead to erroneous and misleading results. Although we used hybrid nanoparticles in our experiments, our results indicate that nanoparticle-based calcium delivery is fundamentally a biochemical phenomenon that is independent of the host animal under investigation.

In the present study, we have shown that calcium-based nanoparticles derived from FBS can be designed to function as a calcium delivery vehicle that disintegrates as a function of pH, and can thus be used to selectively deliver calcium to the acidic wound microenvironment, thereby accelerating cutaneous wound healing. Presumably, this calcium delivery system is not species specific and the calcium-binding proteins used to form the core of our nanoparticles can be derived from sources other than FBS. Future studies will aim to produce species-specific nanoparticles that consist of calcium-binding proteins that are native to the animal species under investigation.

The present study was only designed to investigate the acute effects of nanoparticles, and animals were sacrificed immediately after wound healing was complete. Future studies will be designed to assess the long-term effects of our calcium-based nanoparticles and their impact on tissues outside of the wound bed. The present study provides proof-of-concept that calcium-based nanoparticles can be used as a vehicle to selectively deliver calcium to the acidic wound microenvironment, and thereby enhance cutaneous wound healing. Theoretically, this strategy could be used to selectively deliver other ionically charged payloads to acidic microenvironments.

Although nanoparticles have been studied for over a decade, these are the first experiments to demonstrate that nanoparticles may have a potential therapeutic benefit. Nanoparticle delivery of calcium directly to sites of injury is a novel concept that may be used to effectively improve wound healing. Herein we show that calcium-based nanoparticles can be designed such that they release calcium as a function of pH, but more studies are needed to further optimize this delivery system.
